# Enhancing system stability in power-to-gas applications: integrating biological hydrogen methanation and microbial electrolysis cells under hydrogen overloading in various injection modes

**DOI:** 10.1186/s40643-025-00974-6

**Published:** 2025-11-13

**Authors:** Afrooz Bayat, Ricardo Bello-Mendoza

**Affiliations:** 1 College of Science and Engineering, Flinders University, GPO Box 2100, Adelaide, SA 5001 Australia; 2 Department of Civil and Environmental Engineering, University of Canterbury, Private Bag 4800, Christchurch, 8140 New Zealand

**Keywords:** Biological hydrogen methanation, Microbial electrolysis cells, VFA accumulation, Anaerobic digestion, Power-to-gas (PtG)

## Abstract

Volatile fatty acid (VFA) accumulation is a common issue that compromises the performance of biological hydrogen methanation systems (BHMs). This accumulation is often triggered by fluctuations in hydrogen supply, which can disrupt microbial activity and lead to system instability. To address this challenge, this study investigated the impact of employing a microbial electrolysis cell (MEC) in BHMs to mitigate system instability and acid build-up. As such, a conventional anaerobic digester (AD) and a microbial electrolysis cell, both supplemented with exogenous hydrogen, were evaluated for their performance in hydrogen methanation. The effect of exogenous hydrogen at high addition rates (> 4:1 CO_2_:H_2_ molar ratio) under instantaneous and gradual injection modes was investigated. The results showed that the instantaneous addition of hydrogen resulted in the total failure of the anaerobic digestion system. Propionate accumulated in the system (> 2 g/L) and resulted in low pH (pH = 5.3). Methane production stopped, and the reactor never recovered from hydrogen shock. However, the microbial electrolysis system was able to withstand the instantaneous hydrogen addition and maintain normal operation under toxic hydrogen addition levels (> 4:1 CO_2_:H_2_ molar ratio). Under the gradual injection mode, both MEC and AD reactors remained reasonably unaffected; even though the hydrogen injection exceeded the stoichiometric molar ratio. This study provides a new perspective on the application of MECs for reliable operation and storage of surplus renewable energy via biological hydrogen methanation.

**Graphical abstract:**

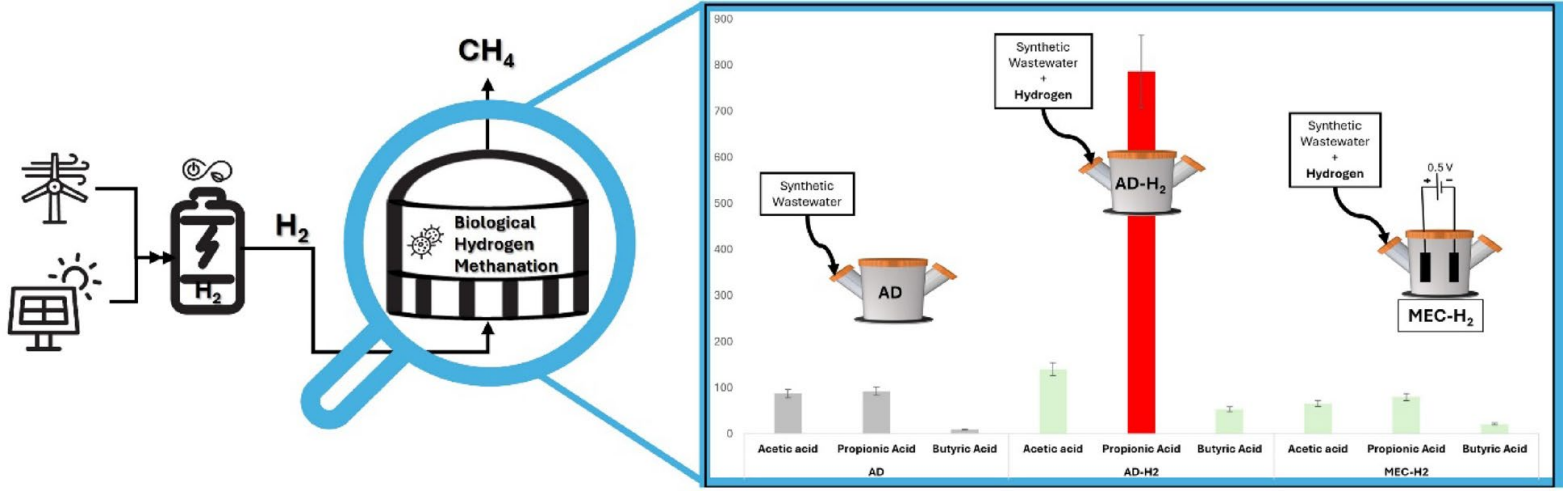

**Supplementary Information:**

The online version contains supplementary material available at 10.1186/s40643-025-00974-6.

## Introduction

The adoption of renewable energy, advancement of hydrogen technologies, and efforts to lower carbon emissions are essential for creating a sustainable and environmentally friendly society (Wei et al. 2025d). Despite their importance, these initiatives face challenges related to large-scale use and efficient storage of energy. To address this, a variety of storage solutions and decarbonization methods have been developed for storing carbon dioxide (Wei et al. 2025b), hydrogen (Wei et al. 2025c), and natural gas (Wei et al. 2025a). As a result, Power-to-gas (PtG) systems have emerged to support sustainability objectives.

The PtG concept not only enables the storage of renewable electricity but also helps balance the supply and demand fluctuations in solar and wind energy systems (Gorre et al. 2020). PtG uses the surplus of electricity generated primarily from Wind and Solar energy systems to green hydrogen via water electrolysis. The green hydrogen is further converted to methane gas via either the Sabatier process or Biological Hydrogen Methanation (BHM). BHM is an approved process by the World Biogas Association (Primmer 2021). In addition, previous research suggests that BHM can be an economical and efficient form of surplus energy storage from renewable electricity production systems (Rusmanis et al. 2019).

In BHM systems, hydrogen is used as a reducing agent in the chemoautotrophic conversion of one mole of carbon dioxide to one mole of methane. Converting H_2_ to methane has gained attention, due to the higher volumetric energy value of methane (36 MJ/m^3^) compared to H_2_ (10.88 MJ/m^3^). An interesting fact about BHM is that, unlike other techniques, there is no unwanted byproduct (Andriani et al. 2014), and while hydrogen eliminates CO_2_ to produce methane gas, any unused hydrogen that is mixed with the final methane gas does not require separation (Bhatia et al. 2020). In fact, the unused mixed hydrogen can increase the combustion energy of the final methane gas as renewable energy known as hythane (Cristiani et al. 2024).

Biological hydrogen methanation occurs primarily in the anaerobic digestion (AD) process, where methane-forming microorganisms, including Acetoclastic Methanogens (AMs) and Hydrogenotrophic Methanogens (HMs), drive the reactions (Kundu et al. 2017). Acetoclastic methanogenic archaea use acetate as their main substrate and convert it to methane, while hydrogenotrophic methanogens directly convert carbon dioxide and hydrogen to methane gas. The latter reaction is thermodynamically more favourable than the former (Alfaro Borjabad 2018). Since this is the primary reaction in BHM systems, it makes the BHM process more attractive. Apart from these primary reactions, Homoacetogens (HAs) also play a key role in AD processes. These microorganisms compete with Hydrogenotrophic Methanogens for hydrogen uptake and convert it to acid in the system. A healthy balance of these microorganisms is essential for the successful operation of the BHM process.

Due to the intermittent nature of renewable electricity input and consequently hydrogen availability, the hydrogen injection into the BHM system can fluctuate significantly. Anaerobic digestion-the core technology of BHMs- is highly sensitive to influent variations, and any fluctuations cause imbalance and disrupt microorganism communities in the system. Variations in hydrogen injection, and more specifically, injecting too much hydrogen, can therefore be counterproductive in BHMs, as it creates a toxic environment in the anaerobic digestion process (Rocamora et al. 2023). It has been reported that a high concentration of hydrogen in anaerobic digestion can inhibit the growth of syntrophic microbes such as propionate degraders and acetate degraders, which leads to acidification of the system (Li 2022; Bensmann et al. 2014). Acidic conditions inhibit methanogenic activity and can negatively affect the methane-producing microorganisms. In this context, a previous study reported that in a BHM system (H_2_-assisted AD system), a high hydrogen content resulted in VFA accumulation due to an increase in hydrolysis and acetogenesis rates (Zheng et al. 2023). In addition, various system integrations have also been used to improve the methanation process. Innovative BHMs, such as a tubular baffled reactor design, were used in a previous study (Savvas et al. 2024). The authors reported major VFA accumulation at 5.5 g/L in their study and described that acetate was the main component of the acid buildup.

In addition, the hydrogen feeding regime is also a key factor for hydrogen uptake and the methanation process (Zhu et al. 2019a). Intermittent feeding is reported to be an efficient technique in hydrogen-assisted AD performance. For example, a previous study (Zhu et al. 2020) explored the addition of hydrogen under intermittent and continuous feeding modes, at rates of 1.5 and 7.2 mL/min, which were set to represent a 1:1 and 4:1 H_2_:CO_2_ molar ratio. The authors observed that the methane content of the reactor increased from 62% (control) to a maximum of 70%. They also observed that the reactor operated under continuous feeding experienced a reduction in methane yield due to a reduction in the abundance of methanogens, specifically acetoclastic methanogens. Eventually, the acetate concentration rose above 1600 mg/L in their study. Various strategies, including pulse addition of hydrogen, the use of a submerged membrane for hydrogen injection, and intermittent and continuous mixing, have also been explored at 4:1 (H_2_:CO_2_) molar ratio or lower (Agneessens et al. 2017; Vechi et al. 2021; Alfaro et al. 2019; Zhu et al. 2019b). The majority of the previous studies focused on hydrogen rates at a maximum molar ratio of 4:1 (H_2_:CO_2_) (Dupnock and Deshusses 2017), which does not reflect the practical availability of hydrogen from the PtG process. As such, the flexibility of the BHMs process for receiving unpredictable or even toxic hydrogen levels (greater than 4:1 molar ratio) (Rocamora et al. 2023; Burke 2001), for the successful operation and control of anaerobic digestion systems remains underexplored.

In summary, despite various attempts by previous studies to improve the performance of BHM, VFA accumulation and system stability, such as the balance between acid production and acid consumption, (Lovato et al. 2016) are still a challenge from an operational view. Many studies have explored hydrogen addition at a 4:1 molar ratio; a comprehensive literature review can be found in the supplementary material section. However, hydrogen availability fluctuates significantly in PtG systems, and a system that is capable of handling molar ratios higher than 4:1 without disrupting the balance between acid and methane production is essential. Therefore, a system with a flexible operational mode is still needed for the successful integration of BHMs into PtG technologies.

Therefore, this research aims to explore the integration of a microbial electrolysis cell into an anaerobic digester as a strategy to enhance the reliability and flexibility of the BHM processes. The study assessed the effect of inhibitory hydrogen concentrations exceeding the stoichiometric 4:1 H_2_:CO_2_ molar ratio, under instantaneous and gradual hydrogen injection, on the performance and stability of the BHM Process.

## Material and methods

Three reactors were set up, each with a working volume of 1.5 L (Table 1). These were glass reactors with a central cap and two side necks for accommodating multiple fittings or sampling ports (Corning® ProCulture®). The reactors were equipped with paddle mixers operated using magnetic stirrers. The glass reactors were then customized accordingly to meet the requirements of each phase of the experiment. The modifications of each glass reactor included:

**Table 1:** Reactor's specification

	AD (with and without H_2_)	MEC
Temperature	Mesophilic (37.5 ℃)	Mesophilic (37.5 ℃)
Reactor volume	3L	3L
Working volume	1.5L	1.5L
Organic carbon source	Glucose	Glucose
Influent concentration	10 g/L	10 g/L
External voltage	–	0.5 V
Electrodes	–	× 3 Carbon brush (anode) × 3 carbon cloth (cathode)

1.Hydrogen-assisted anaerobic digestion: this reactor had all the baseline specifications of the conventional anaerobic digestion reactor, with an additional port connected to a gas diffuser positioned at the reactor base to facilitate hydrogen dispersion.

2.Microbial Electrolysis Cell: The MEC used in this experiment had the same features as the hydrogen-assisted AD with an additional set of electrodes inserted through the reactor’s central cap. The electrodes were then connected to a power supply.

The MEC was constructed using a single-compartment glass bottle, retrofitted with three anodes and three cathodes. The electrodes were interconnected with titanium wire. The cathode consisted of a 5 × 5 cm^2^ carbon cloth coated with 0.5 mg/cm^2^ of 60% Platinum on Vulcan (carbon black). The anode was a carbon brush (2 cm diameter × 3 cm brush-section × 10 cm overall length; titanium stem; PX35 carbon fiber fill with 400,000 tips per inch). A top view of the MEC reactor configuration is provided in the Supplementary Material.

The power supply was custom-built at the University of Canterbury. It provided an adjustable voltage source capable of delivering up to 2 A of current. It enabled direct measurement of the voltage across the cell, the output current, and an internal voltage reference point for data logging via UDL. Additionally, the power supply and data acquisition system were configured to alert the user if the output current reached 2 A, and to automatically reduce the output voltage to maintain the current at 2A for safety. The cell voltage was maintained at 0.5 V between the cathode and anode terminals. The electrolyte consisted of 50 mM phosphate buffer (K_2_HPO_4_ and KH_2_PO_4_) and nutrients to support biomass growth, formulated at a COD: N:P ratio of 250:5:1.

Each reactor was connected to a gas flow meter (Bioprocess Control, Sweden), and the produced gas was collected in a Tedlar gas bag. For standard biogas (without hydrogen), the gas was collected in a 1-L Tedlar bag, while during the hydrogen injection phase, a 1-L Flexfoil bag (SKC) was used to ensure hydrogen impermeability. Gas samples were taken using a gas-tight syringe.

To ensure anaerobic conditions, the reactors were purged with pure nitrogen gas for one minute at the start of the experiment to remove residual oxygen and then sealed with silicone. To monitor the anaerobic environment and any oxygen presence, the headspace was regularly analyzed using a landfill gas monitor (GA5000, Geotech). All systems were maintained in a temperature-controlled room under mesophilic conditions at 37.5 °C.

When required, hydrogen was injected to the reactors via a mass flow controller (ALICAT©) connected to a hydrogen gas cylinder. Two timer valves were integrated with the mass flow controller to direct hydrogen flow to the reactors. The valve open times were controlled via a computer interface using a custom-built control box developed at the University of Canterbury.

To ensure laboratory safety, ambient hydrogen levels were monitored using a hydrogen detector (Honeywell BW™ Solo; 0–1000 ppm range, 2 ppm resolution) equipped with a built-in alarm system, which was placed in the temperature-controlled room.

### Gradual vs. Instantaneous hydrogen injection protocols

The performance of the MEC under inhibitory hydrogen concentrations exceeding stoichiometric molar ratios was evaluated and compared to that of the anaerobic digester, which served as a control. The experiment was conducted in two phases, instantaneous (phase I) and gradual (phase II) hydrogen addition, to assess whether the hydrogen addition mode affected the performance of the reactors when hydrogen was above stoichiometric ratios.

#### Instantaneous hydrogen addition

In phase I, hydrogen was added instantaneously into the systems (i.e., in a single daily injection), starting with 100 mL H_2_/day. The volume of hydrogen injected was increased daily in steps of 100 mL/day until it reached 500 mL/day on the fifth day. Subsequently, hydrogen addition remained constant at instantaneous injections of 500 mL/day for the duration of phase I.

#### Gradual hydrogen addition

In phase II, a new set of experiments was conducted, with the reactors receiving the same total volume of hydrogen as in the previous step (i.e., 500 mL/day) but following a gradual injection regime. In this phase, hydrogen was supplied at a rate of 4 mL/min for approximately 2 h per day to each reactor. A conventional AD without hydrogen addition was included to collect baseline data.

#### Inoculum and wastewater feed

Acclimated MEC-H2 from a previously operated reactor at the University of Canterbury was used as the microbial inoculum for this phase. The reactors had been operated for over a year, resulting in the formation of a well-established biofilm on the electrodes. The feed composition remained consistent throughout the duration of the study. The anaerobic digestion reactors (AD and AD-H2) were also carried over from a previously acclimated system. All reactors were initially seeded with mesophilic anaerobic sludge (14.4 g VS/L) obtained from Bromley’s wastewater treatment plant in Christchurch, New Zealand. At the start of the operation, the inoculum was mixed with an anaerobic medium following procedures described in previous studies (Angelidaki and Sanders 2004).

Synthetic wastewater was prepared with a C/N ratio of 20 (Table 2) to support healthy microbial growth (Xiao et al. 2022). This ratio typically resembles food industry wastewater, which contains high entrained energy at approximately 10 g/L COD (Carrillo-Reyes et al. 2019). All reactors were operated as continuous stirred tank reactors (CSTR) using magnetic stirrers, with a hydraulic retention time (HRT) of 30 days. Hydraulic retention time was calculated as the ratio of the reactor’s working volume to influent flow rate, corresponding to 30 days for all the reactors, including the microbial electrolysis cells (MECH2). Accordingly, the organic loading rate (OLR) was calculated as the COD influent concentration divided by the hydraulic retention time, resulting in an OLR of 0.33 g COD/L.day for all reactors.

**Table 2:** Synthetic wastewater

Chemical compound	Unit	Concentration
C_6_H_12_O_6_	g/L	9.37
(NH_4_)_3_PO_4_	mg/L	764.15
CoCl_2_·6H_2_O	mg/L	1.2
FeCl_2_·4H_2_O	mg/L	7.8
MnCl_2_·4H_2_O	mg/L	0.376
Na_2_MO_4_·2H_2_O	mg/L	0.375
NiCl_2_·6H_2_O	mg/L	0.45

The reactors followed a typical anaerobic digestion configuration in a single-phase, single-chamber-type system, where acidogenesis and methanogenesis occurred within the same reactor.

### Testing protocols and analytical methods

During the experiment, water and gas quality parameters were tested, including chemical oxygen demand (COD), volatile fatty acid (VFAs), pH and biogas composition.

#### Chemical oxygen demand (COD)

To measure the soluble COD, influent and effluent samples were filtered through a 0.45-µm syringe filter. Two mL of the samples were mixed with 5 mL of the digestion solution (Hach TNT 822) and added to COD vials. The digestion solution contained an oxidising agent (potassium dichromate), a catalyst to accelerate the reaction, and mercury sulphate to mask the effect of the chloride ion on the results. The COD test included a 2-h digestion period at 105 °C that was done using a closed reflux digestion unit (Digital reactor, block 200, HACH). After digestion, the COD value (mg/L) was measured using a HACH Digital DR 3900 spectrophotometer at a wavelength of 620 nm. A standard solution of potassium hydrogen phthalate (KHP) was used for quality control of the test results with a standard COD level of 600 mg/L.

To quantify COD accurately, a series of standard solutions was prepared using KHP as the reference compound. These standards covered a range of concentrations relevant to the expected sample values. Each standard was analyzed following the procedures described above. The absorbance values obtained were plotted against the theoretical COD concentrations to generate a calibration curve. A linear regression model was applied, and then the resulting equation was used to determine the COD of unknown samples by fitting their absorbance values to the curve.

#### pH

The reactors’ pH was measured daily using a pH electrode (RE 357 Tx Microprocessor pH Meter). To calibrate the pH meter, reference solutions with acidic (pH = 4), alkaline (pH = 10), and neutral (pH = 7) pH values were used according to the manufacturer’s instructions.

#### Volatile fatty acids (VFAs)

Samples for VFA analysis were collected from effluent tabs every 24 h (i.e. once every feeding cycle). A 0.22-μm glass fibre filter was used for sample filtration. The samples were subsequently acidified with concentrated phosphoric acid, adjusting the pH to approximately 2 prior to analysis. A gas chromatograph (Nexis GC-2030, Shimadzu, Japan) was used for measuring the amount of Acetic acid (HAc), propionic acid (HPr), and butyric acid in either iso or n-butyric form (HBu) in the samples. The gas chromatograph was equipped with a flame ionization detector (FID) and a capillary column (30 m × 0.25 mm × 0.25 μm; HP-INNOWAX USA). Operating conditions included a column temperature of 120 °C, an inlet pressure of 154.2 Kpa, and a column flow rate of 1.66 ml/min. The flow rates of H_2_, air, and N_2_ in the GC-FID were 32, 200, and 24 ml/min, respectively. The identification of volatile fatty acids (acetic, propionic, and butyric) was based on comparing retention times with corresponding standards (2.9, 3.5, and 4.2 min, respectively). Regular calibration of the GC system and validation of result accuracy were performed using standard solutions of acetic acid, propionic acid, and butyric acid at varying concentrations.

VFA calibration was performed using standard solutions of individual acids, including acetic, propionic and butyric acids, at varying concentrations. These standards were analyzed using gas chromatography (GC). The peak areas corresponding to each acid concentration were recorded and plotted against the respective concentration to construct calibration curves. Unknown samples were injected into the GC, and the resulting peak areas were used to determine the concentration of individual VFAs by referencing the appropriate calibration curves. In addition, Deionized water was injected after every 5 samples to check the accuracy of measurements.

#### Biogas volume and composition

Biogas volume was measured using gas flowmeters (Bioprocess Control model µFlow) every 24 h (once every feeding cycle). Biogas composition was analyzed using a gas chromatograph equipped with a thermal conductivity detector (GC-TCD) (Agilent 7820A, China). Four-mL gas samples were injected into the GC-TCD to determine the gas composition. The GC-TCD was fitted with an Agilent 19095P-Q04 stainless steel column with 30 m × 530 μm ~ 40 μm. Helium was used as the carrier gas at a flow rate of 10 mL/min and a pressure of 10.6 psi. The oven temperature was maintained at 30 °C, the injector temperature at 70 °C, and the detector temperature at 155 °C. The retention times of nitrogen, methane and carbon dioxide were 1.44, 1.59, 2.12 min, respectively. A standard gas mixture of 30% CO_2_, 10% N_2_, and 60% methane was used regularly to check the accuracy of results.

Calibration for biogas composition analysis was conducted using certified gas mixtures containing known concentrations of methane and carbon dioxide. These standards were analyzed with the GC-TCD. The detector response (peak area) for each gas was plotted against its certified concentration to generate individual calibration curves. The linear regression equations derived from these curves were used to calculate the concentrations of gases in unknown samples based on their GC peak areas. Calibration was repeated periodically to ensure accuracy and instrument stability.

### System stability metrics

To evaluate the system’s stability under different hydrogen addition modes, several key parameters were selected and monitored throughout the experiments. The volatile fatty acids (VFA) concentration was identified as the primary indicator of system imbalance, since VFA accumulation occurs due to an imbalance between biological reactions in anaerobic digestion processes. pH fluctuations were also monitored as they are closely related to VFA buildup. A drop in pH shows an imbalance between acidogenesis and methanogenesis phases, as well as a lack of sufficient alkalinity. Methane production rate was included as a third critical parameter, given that it is directly sensitive to both VFA concentration and pH levels. The coefficient of variation (CV) of data collected for each of these parameters was used to evaluate process stability quantitatively. A CV below 15% was considered indicative of low variability and, therefore, stable system performance (Fisgativa et al. 2016).

### Statistical analysis

A one-way analysis of variance (ANOVA) was conducted to assess the effect of the system configuration, namely Conventional Anaerobic Digestion (AD), Hydrogen-assisted Anaerobic Digestion (ADH2) and Hydrogen-assisted Microbial Electrolysis Cells (MECH2), operating under gradual and instantaneous hydrogen addition, on the dependant variables: biogas production, methane content, methane yield, pH, effluent propionate and effluent acetate. In addition, a Levene’s test was conducted to assess the assumption of equal variances across three different reactors (AD, ADH2, MECH2) under two hydrogen addition modes. A one-way Welch’s ANOVA was also conducted to determine if there were significant differences between employing three different reactors when the assumption of equal variances was violated. Post-hoc tests (Games-Howell) were conducted to identify which groups differed significantly from one another.

The results section only discusses the results when a significant difference was identified between AD, ADH2 and MECH2 under gradual and instantaneous hydrogen addition modes on the dependent variables. A detailed description of the statistical analysis tests is provided in the supplementary material accompanying the output of one-way ANOVA tests. The descriptive analysis in the Supplementary Material includes the number of samples in each group (n), mean (M), Standard deviation (SD), Levene Statistic value, degrees of freedom, eta-squared, and p-values of Post Hoc (Games-Howell) test.

## Results and discussion

### Biogas production and methane content under instantaneous and gradual hydrogen addition

**Fig. 1:**
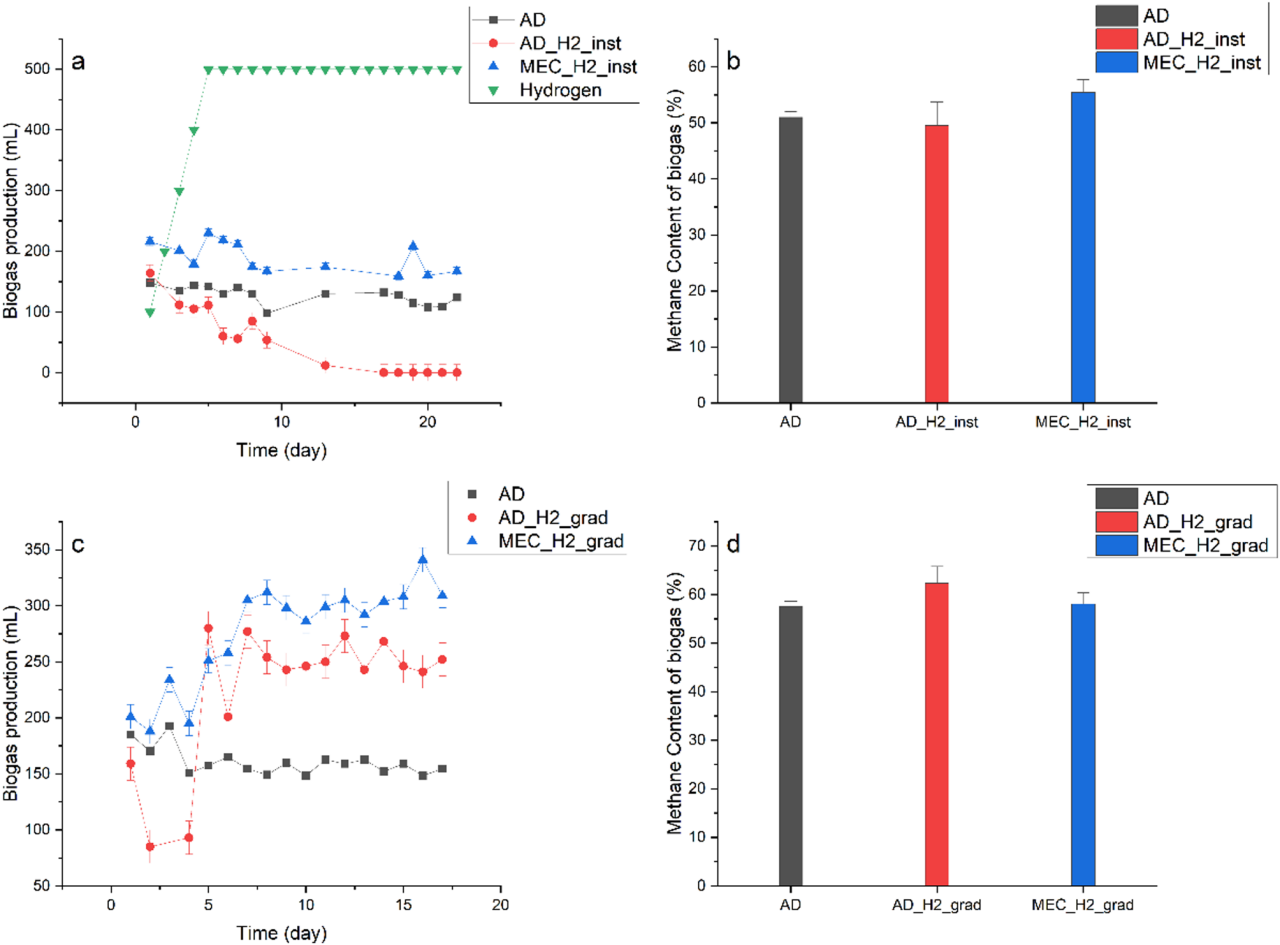
**a** daily biogas production in Phase I (instantaneous hydrogen addition); **b** Percentage of methane gas in the biogas (%) in Phase I(instantaneous hydrogen addition); **c** daily biogas production in Phase II (gradual hydrogen addition); **d** Percentage of methane gas in the biogas (%) in Phase II (gradual hydrogen addition)

As can be seen in Fig. 1a, immediately after the commencement of hydrogen addition in Phase I, biogas production in both reactors (ADH2_inst and MECH2_inst) started declining until the end of day five. After day five, MECH2_inst started recovering from the hydrogen shock and resumed biogas production at an average level of 189.53 ± 6.65 mL/day, which was higher than that of the conventional AD at 127.53 ± 3.59 mL/day (*P* < 0.001). However, biogas production continuously decreased in the ADH2_inst until there was no biogas production after day 13, and the reactor stayed inactive for the rest of Phase I and never recovered from the hydrogen overloading.

MECs have a great capacity to provide a robust environment by favouring the growth of microorganisms that are resilient to high concentrations of toxic substances, including sodium, acetate, ammonium, and hydrogen (Meegoda et al. 2018)*.* AD’s microbial balance is a key factor for stable performance of the process; therefore, a more robust and healthy community of microorganisms results in enhanced productivity of the AD process under toxic conditions. As such, the results of the current study showed that integrating MEC with AD improves the overall performance (*P* < 0.001) of the system and alleviates the system’s upset likely by proliferating a more robust community of microorganisms. In a previous study employing an MEC with an AD process resulted in a more diverse and resilient community of microorganisms (Bayat and Bello-Mendoza 2025) that improved the overall performance of anaerobic digestion process. In this study, when hydrogen was used for BHM in the AD reactors, higher concentrations of hydrogen in the system and its instantaneous injection led to system failure except in the MECH2_inst. This showed that MEC maintained the balance of acid-producing and methane-producing processes in the BHM systems.

As Fig. 1b shows, after the instantaneous hydrogen addition started, the methane content of the hydrogen-assisted reactors followed a continuous but moderate increase pattern until day seven, when the methane content increased by 7.8% in the MECH2_inst and by 3.6% in the ADH2_inst (*P* < 0.001). After seven days, MECH2_inst reached a plateau and remained at a constant methane content of approximately 57%. However, after seven days of slow increase in methane content in ADH2_inst, methane content of the biogas decreased sharply until the end of Phase I. Eventually, when the reactor stopped biogas production, biogas content monitoring was ceased. In line with what was observed in this study, Guo et al. (2024) stated that methane production can have a direct correlation with VFA accumulation in the system.

In phase II of the experiment, the systems were overloaded with hydrogen; however, unlike phase I, instead of instantaneous hydrogen injection, the injection was conducted gradually at a rate of 4 mL/min. However, hydrogen addition was still at 500 mL/day, resulting in a hydrogen concentration of > 4:1 molar ratio. Upon addition of hydrogen, the biogas production in the MECH2_grad reactor increased quickly and remained at an average of 275.64 ± 10.86 mL/day. Comparing this value with that of phase I, when the hydrogen was added in a single injection, it shows that gradual hydrogen injection to MEC reactor resulted in higher biogas production (Fig. 1c).

The ADH2_grad reactor showed moderate fluctuations in biogas production in the first few days of Phase II; however, it soon recovered and maintained a stable biogas production throughout the rest of this phase. In phase II, more biogas production was observed for AD-H_2_ compared to Phase I, with an average level of 225.68 ± 14.76 mL/day, which shows the importance of the hydrogen addition mode in the BHM systems.

With regards to the hydrogen injection mode—i.e., instantaneous vs. gradual injection of hydrogen—the ADH2_inst system was affected the most. It was interesting to observe that, although in both cases the hydrogen volume was at toxic levels, gradual hydrogen addition to the ADH2_grad reactor did not result in the failure of the system. This may be due to the fact that a gradual addition of hydrogen allows microorganisms sufficient time for the utilisation of H_2_ and other intermediate substances such as acetate and propionate. In contrast, instantaneous hydrogen addition cripples and shocks microorganisms, leading to system failure. According to Alfaro Borjabad (2018) and Agneessens et al. (2017), gradual hydrogen addition can allow more time for the microorganisms to utilize the hydrogen. This can result in a quicker recovery from any potential toxicity of hydrogen posed to the BHM systems in this research.

In phase II, the methane content remained stable, indicating consistent biogas production. The methane percentage of both MECH2_grad (58.04 ± 2.27, *P* < 0.001) and ADH2_grad (61.0176 ± 0.53%, *P* =  < 0.001) improved compared to AD (Fig. 1d). However, the statistical analysis did not show a significant difference between MECH2_grad and ADH2_grad (*P* = 0.262). The CO_2_ percentage in the reactors was also at 39.25 ± 0.4, 25.74 ± 1.1, 25.43 ± 0.77 in AD, ADH2_grad, MECH2_grad, respectively. While a few studies have reported high methane concentrations, the majority of the literature indicates that the methane content of biogas typically ranges between approximately 50% and 60% (Dupnock and Deshusses 2017).

Although the MECH2_grad reactor produced biogas with a lower methane percentage compared to ADH2_grad, its higher overall biogas production resulted in a greater total volume of methane. Although this result was unexpected, existing literature indicates that reduced methane concentration in biogas is not uncommon under conditions of elevated methane production (De Albuquerque et al. 2020).

### System stability under instantaneous vs. gradual hydrogen addition: methane yield, pH fluctuations and VFA accumulation

#### Methane yield

**Fig. 2:**
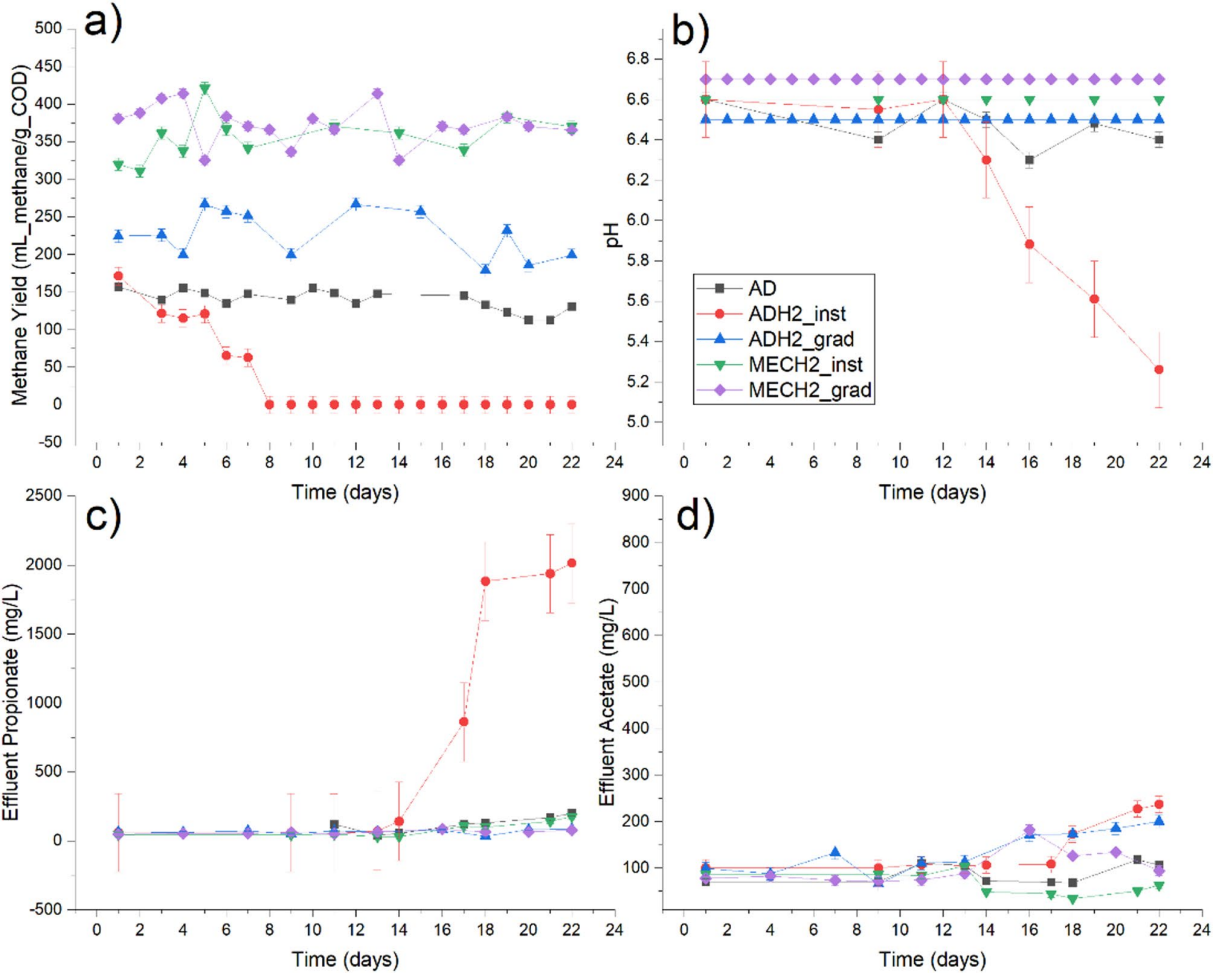
**a** comparative analysis of daily methane yield under gradual (grad) and instantaneous (inst) hydrogen addition; **b** pH variations during Phase I and Phase II; **c** effluent propionate in the reactors under hydrogen addition modes; **d** effluent acetate of the reactors under hydrogen addition modes

At the start of Phase I (instantaneous hydrogen addition), ADH2_inst showed a higher methane yield compared to the control system (AD). However, this value consistently decreased over time, eventually dropping below the methane yield of AD (*P* < 0.001). As depicted in Fig. 2a, the methane yield in ADH2_inst gradually declined to zero, and the system stopped converting organic material to methane gas. In contrast, gradual hydrogen addition enhanced ADH2_grad methane yield compared to the control system (*P* < 0.001). Despite moderate fluctuations in methane yield, ADH2_grad consistently operated at an enhanced level throughout the experiment. According to Fig. 2a, hydrogen addition mode barely impacted the MEC performance, where both MECH2_inst and MECH2_grad showed a similar trend in methane yield (mL methane/gCOD). This suggests that methane production followed an electrochemical pathway where CO_2_ and H_2_ were directly converted to methane gas at the cathode; a complete description of the reaction pathways in the MEC can be found elsewhere (Bayat and Bello-Mendoza 2025). Additionally, MECs promote acetoclastic methanogenesis pathway where sufficient provision of electrons at the anode facilitates the electrochemical conversion of acetate to methane gas. This combined pathway of electrochemical systems explains the higher methane yield in the MECH2 compared with the ADH2. This finding has significant implications as it supports the practical deployment of BHM systems using MECs. This means that regardless of hydrogen availability and hydrogen injection mode, even under toxic hydrogen concentrations, MECs can successfully facilitate hydrogen-to-methane conversion for power-to-gas applications. This enhances operational simplicity, which is a key criterion for adapting PtGs technology.

#### pH variations

To better understand system stability, pH variations were analyzed. The pH level was monitored since it is an essential indicator of a well-functioning anaerobic digestion system (Kullavanijaya and Chavalparit 2022). In Phane II, all reactors showed a stable pH level at an average level of 6.7, 6.6 and 6.5 for the MECH2_grad_,_ AD and ADH2_grad, respectively (Fig. 2b). The differences between ADH2_grad and AD were insignificant (*P* = 0.744). However, the statistical analysis showed significant differences between MECH2_grad and the other two systems (ADH2_grad (*P* < 0.001) and AD (*P* = 0.004)).

For optimal methanogenic activity, a healthy pH range of 6.5–7.5 is required in anaerobic digestion systems. The MECH2___inst maintained a healthy pH level of 6.6 (Fig. 2b). Although the pH level was in the lower range of the recommended value in all reactors, it was still acceptable for proper methanogenic activity. The observed low pH even in the MECH2_inst is attributed to the high degradability of the organic carbon (glucose) in the feed, which caused the rapid degradation of organic carbon to VFA and consequently resulted in lower pH ranges. The MECH2_inst and AD reactors maintained relatively constant pH; notably, the MECH_2__inst exhibited the most consistent pH level throughout the experiment, even under shock conditions. This is noteworthy as maintaining a stable pH level is a key factor in the successful operation of BHMs.

In case of ADH2_inst, the pH started dropping since day 12 of the experiment and continued decreasing until it reached pH = 5.3, which is well below the recommended value for optimal pH for AD processes (Liu et al. 2024). The ADH2_inst reactor was maintained and cared for after the experiment for longer HRTs to stimulate the syntrophic acetate oxidation and hydrogenotrophic methanogens as stated in previous research (Wang et al. 2018); however, it never recovered from the hydrogen shock, and the pH remained low, which eventually led to reactor souring.

#### VFA Accumulation

In line with the observed pH profile under gradual hydrogen addition, no major VFA accumulation was detected in any of the reactors. The VFA trend is shown in Fig. 2b and c. Notwithstanding, the ADH2_grad reactor had the highest acetate and propionate concentration in the effluent at an average level of 142.1 ± 11.4, and 86.48 ± 2.5 mg/L, respectively. The lowest acetate and propionate concentrations were observed in the MECH2_grad, with average levels of 95.97 ± 11.56 and 61.8 ± 3.69 mg/L, respectively. In any case, the VFA levels were below the inhibitory thresholds (Liu et al. 2022) and did not negatively affect the performance of the reactors. The statistical analysis also showed that there is no significant difference between the effluent acetate and propionate of any of the reactors since the P-values were always greater than 0.05 in all cases (more information is provided in the Supplementary Material). In general, the improved VFA reduction in MEC is attributed to the voltage supply that provides ample electrons for direct electrochemical VFA oxidation at the anode (Park et al. 2020). According to Guo et al. (2013), VFA and COD conversion correlates directly with the applied voltage in the microbial electrolysis cells, meaning that the applied voltage can improve the destruction of VFAs and COD in the system. In the current study, the MEC also showed these characteristics even under a hydrogen concentration at toxic levels. This means that although hydrogen could negatively affect the acetate and propionate degraders in the AD system, MEC’s capability in accelerating anodic VFA oxidation alleviated VFA build-up in the system. This is explained by the shift in the direction of biological reactions towards the oxidation of acetate by electrogenic bacteria (Cheng et al. 2009).

Similarly, under instantaneous hydrogen addition, VFA concentrations in MECH2_inst remained relatively stable, with no significant accumulation detected. Although moderate levels of acetic acid and propionic acids were detected in the system’s effluent, the VFA concentration was well below the threshold for anaerobic digestion systems. It was interesting to see that the addition of hydrogen to the MECH2_inst reactor caused a slight decrease in the effluent acetate concentration from a baseline of 104 mg/L to a minimum of 33.65 mg/L. This suggests that coupling MECs with anaerobic digestion can promote further acid removal, which is an essential requirement for BHM systems. Effluent butyric acid concentration was observed to be insignificant in the systems studied.

In contrast, effluent propionic acid concentration in ADH2_inst escalated considerably from safe ranges to approximately 2000 mg/L, which is well above recommended values for the anaerobic digestion process. VFA accumulation persisted in the system, and methane production decreased until no more VFA degradation or methane production was observed in the ADH2_inst system. Propionic acid has been observed as the main contributor compound in the VFA accumulation of the AD systems (Yang et al. 2025), which is in line with the findings of the research reported here. ADH2_inst was unable to tolerate the instantaneous hydrogen overload at toxic concentrations, resulting in reduced VFA degradation and a subsequent drop in pH, which ultimately led to system failure. This experimental finding aligns with theoretical models from previous studies (Bensmann et al. 2014), where a BHM model predicted that elevated hydrogen concentrations have inhibitory effects on anaerobic digestion. This inhibition leads to a decrease in pH, which in turn suppresses acid-degrading microbial activity, resulting in system acidification and the washout of the propionate-degrading microorganisms. Similarly, in the current study, hydrogen overload at toxic concentrations consequently resulted in propionate accumulation in ADH2-inst followed by system acidification. System acidification suppressed methanogenic activity, and methane production ceased entirely (Wang et al. 2019; Chen et al. 2020).

## Limitations

While the findings of this study contribute valuable insights for BHM operation, the study is subject to limitations that warrant consideration. Firstly, the study was conducted under controlled laboratory conditions, and further research is needed to assess the scalability and applicability of the findings to industrial settings. Furthermore, future work should explore the performance of the proposed system with real wastewater to test its practical application for the BHM process. Additionally, expensive platinum was used as the cathodic catalyst in this study. For practical scaling up of this setup, future studies should focus on more cost-effective cathodic catalysts in place of platinum. Lastly, the microbial community composition was not analyzed. Understanding the microbial dynamics could provide deeper insights into system resilience and BHM efficiency. Incorporating metagenomic or microbial profiling techniques in future work would strengthen the biological interpretation of the results.

## Conclusion

The effect of hydrogen overloading and injection modes on the performance of biological hydrogen methanation systems were evaluated. In addition, the effect of hydrogen overloading on the performance of an integrated MEC in BHM reactors was explored. The experimental analysis showed that the anaerobic digestion process can entirely fail upon instantaneous addition of hydrogen. Overloading the anaerobic digestion with instantaneous and pure hydrogen addition resulted in major VFA accumulation in the form of propionic acid, with VFA concentrations above 2000 mg/L in the reactor, and a sharp drop in pH to 5.

Regardless of the hydrogen injection mode, the integrated MEC reactor demonstrated better performance at high hydrogen concentrations (levels considered toxic to the AD microbes). More importantly, an improvement was observed in methane production, VFA and COD destruction and pH stability, therefore confirming the superiority of the MEC reactor in the BHM processes.

Overall, although the methane production in the integrated MEC reactor decreased for a short time, the reactor was able to recover from the hydrogen shock without any control and remained stable for the rest of the experiment. This finding could contribute to simplifying BHM processes where pure hydrogen is used for storage in the form of methane gas. Therefore, employing an MEC in the BHM plants can alleviate any negative hydrogen injection variation, mainly because MEC was found to be tolerant of high hydrogen addition and balancing VFA production and utilisation, which is the key factor in stabilising the BHM process.

## Supplementary Information

Below is the link to the electronic supplementary material.

Supplementary Material 1

## Data Availability

The datasets generated during and/or analysed during the current study are available from the corresponding author on reasonable request.

## Abbreviations

PtG: Power-to-gas
VFA: Volatile fatty acids
BHMs: Biological hydrogen methanation systems
MEC: Microbial electrolysis cell
AD: Anaerobic digestion
AMs: Acetoclastic methanogens
HMs: Hydrogenotrophic methanogens
HAs: Homoacetogens
CSTR: Continuous stirred tank reactors
HRT: Hydraulic retention time
OLR: Organic loading rate
COD: Chemical oxygen demand
GC: Gas chromatography
CV: Coefficient of variation
MECH2: Hydrogen-assisted microbial electrolysis cells
ADH2: Hydrogen-assisted anaerobic digestion
