# Supplementary material for: Enhancing system stability in power-to-gas applications: integrating biological hydrogen methanation and microbial electrolysis cells under hydrogen overloading in various injection modes
Source: Bioresour Bioprocess. 2025 Nov 13;12(1):135. doi: 10.1186/s40643-025-00974-6 (PMC12615864; doi:10.1186/s40643-025-00974-6)
Supplement: Supplementary file 1 — Supplementary Material 1 [file 40643_2025_974_MOESM1_ESM.docx]

Supplementary Materials

# Article Title

# Enhancing System Stability in Power-to-Gas Applications: Integrating Biological Hydrogen Methanation and Microbial Electrolysis Cells under Hydrogen Overloading in Various Injection Modes

# Authors:

Afrooz Bayat^a,*^, Ricardo Bello-Mendoza^b^

^a^ College of Science and Engineering, Flinders University, GPO Box 2100, Adelaide SA 5001, Australia [Afrooz.bayat@flinders.edu.au](mailto:Afrooz.bayat@flinders.edu.au)

^b^ Department of Civil and Environmental Engineering, University of Canterbury, Private Bag 4800, Christchurch 8140, New Zealand, [ricardo.bellomendoza@canterbury.ac.nz](mailto:ricardo.bellomendoza@canterbury.ac.nz)

***corresponding author:**

Afrooz Bayat

Email: [Afrooz.bayat@flinders.edu.au](mailto:Afrooz.bayat@flinders.edu.au)

**Tables:**

[Table 1 One-way ANOVA results 2](#_Toc209439555)

[Table 2 Comparison of Reactor configuration and results of biological hydrogen methanation system 23](#_Toc209439556)

**Figures:**

[Figure 1 One-way ANOVA results - Effluent acetate under gradual hydrogen addition 9](#_Toc209439535)

[Figure 2 One-way ANOVA results - Effluent acetate under instantaneous hydrogen addition 10](#_Toc209439536)

[Figure 3 One-way ANOVA results - Biogas production under instantaneous hydrogen addition 11](#_Toc209439537)

[Figure 4 One-way ANOVA results Biogas production under instantaneous hydrogen addition 12](#_Toc209439538)

[Figure 5 One-way ANOVA results - Methane content under gradual hydrogen addition 13](#_Toc209439539)

[Figure 6 One-way ANOVA results - Methane content under instantaneous hydrogen addition 14](#_Toc209439540)

[Figure 7 One-way ANOVA results - Methane yield under gradual hydrogen addition 15](#_Toc209439541)

[Figure 8 One-way ANOVA results - Methane yield under instantaneous hydrogen addition 16](#_Toc209439542)

[Figure 9 One-way ANOVA results - pH variations under gradual hydrogen addition 17](#_Toc209439543)

[Figure 10 One-way ANOVA results - pH variations under instantaneous hydrogen addition 18](#_Toc209439544)

[Figure 11 One-way ANOVA results - Effluent propionate under instantaneous hydrogen addition 19](#_Toc209439545)

[Figure 12 One-way ANOVA results - Effluent propionate under gradual hydrogen addition 20](#_Toc209439546)

[Figure 13 Microbial Electrolysis cell reactor top-view showing configuration of anodes and cathodes 21](#_Toc209439547)

[Figure 14 Methane yield (MEC with and without Hydrogen) 22](#_Toc209439548)

Table 1 One-way ANOVA results

| **Dependant Variable** | **Hydrogen addition mode** | ***Results*** |
| --- | --- | --- |
| Biogas Production | Gradual | *This study compared the effects of various systems on biogas production under gradual hydrogen addition. AD (n=15), ADH2_grad (n=15), MECH2_grad (n=15)*  *Mean test scores and standard deviations were:*   - *AD (M = 160.7206, SD = 12.35158).* - *ADH2_ grad (M = 225.6875, SD = 60.99805).* - *MECH2_ grad (M = 275.6471, SD = 46.17215).*   *We conducted a one-way ANOVA to compare the means of the three groups.*  *A one-way ANOVA revealed a significant effect of the reactors on biogas production under instantaneous hydrogen addition, F(2,* *47) = 9.181, p < 0.001. The effect size, eta squared (η²), was 9.181, indicating a large effect.*  *Games-Howell post hoc test showed the MECH2_grad scored higher than both AD (p < 0.001) and ADH2_grad (p =0.035) groups. The ADH2_grad reactor scored higher than the AD (p =0.002).*  *These findings suggest that MECH2_inst leads to the highest biogas production under gradual hydrogen addition, followed by ADH2_grad, and lastly, AD. The effect size confirms these differences are practically significant.* |
| Biogas Production | Instantaneous | *This study compared the effects of various systems on biogas production under instantaneous hydrogen addition. AD (n=15), ADH2_inst (n=15), MECH2_inst (n=13)*  *Mean test scores and standard deviations were:*   - *AD (M = 127.5333, SD = 14.43145).* - *ADH2_inst (M = 50.6000, SD = 54.38067).* - *MECH2_inst (M = 189.5385, SD = 24.99205).*   *We conducted a one-way ANOVA to compare the means of the three groups.*  *A one-way ANOVA revealed a significant effect of the reactors on biogas production under instantaneous hydrogen addition, F(2,* *40) = 15.248, p < 0.001. The effect size, eta squared (η²), was 0.724, indicating a large effect.*  *Games-Howell post hoc test showed the MECH2_inst scored higher than both AD (p < 0.001) and ADH2_inst (p < 0.01) groups. The AD reactor scored higher than the ADH2_inst (p < 0.05).*  *These findings suggest that MECH2_inst leads to the highest biogas production under instantaneous hydrogen addition, followed by AD, and lastly, ADH2_inst. The effect size confirms these differences are practically significant.* |
| Methane content | Gradual | *This study compared the effects of various systems on methane content under gradual hydrogen addition. AD (n=9), ADH2_grad (n=11), MECH2_grad(n=11)*  *Mean test scores and standard deviations were:*   - *AD (M = 50.9298, SD = 1.10877).* - *ADH2_ grad (M = 61.0176, SD = 5.53884).* - *MECH2_ grad (M = 58.0455, SD = 2.27941).*   *We conducted a one-way ANOVA to compare the means of the three groups.*  *A one-way ANOVA revealed a significant effect of the reactors on biogas production under gradual hydrogen addition, F(2,* *28) = 4.84, p =0.016. The effect size, eta squared (η²), was .586, indicating a large effect.*  *Games-Howell post hoc test showed the AD scored lower than both ADH2_grad (p < 0.001) and MECH2_grad (p < 0.001) groups. There was no meaningful difference between the methane content of ADH2_grad reactor and MECH2_grad (p =0.262).* |
| Methane content | Instantaneous | *This study compared the effects of various systems on biogas production under instantaneous hydrogen addition. AD(n=18), ADH2_inst (n=12), MECH2_inst (n=18)*  *Mean test scores and standard deviations were:*   - *AD (M = 50.9610, SD = 1.02953).* - *ADH2_inst (M = 49.4881, SD = 4.26884).* - *MECH2_inst (M = 55.4526, SD = 2.25998).*   *We conducted a one-way ANOVA to compare the means of the three groups.*  *A one-way ANOVA revealed a significant effect of the reactors on methane under instantaneous hydrogen addition, F(2, 45) = 6.956, p = 0.002. The effect size, eta squared (η²), was .501, indicating a large effect.*  *Games-Howell post hoc test showed the MECH2_inst scored higher than both AD (p < 0.001) and ADH2_inst (p = 0.001) groups. The results show that there’s no meaningful difference between AD and ADH2_inst reactor (p=0.491)*  *These findings suggest that MECH2_inst leads to the highest methane content of biogas under instantaneous hydrogen addition, followed by AD, and lastly, ADH2_inst. The effect size confirms these differences are practically significant.* |
| Methane yield | Gradual | *This study compared the effects of various systems on methane under gradual hydrogen addition. AD (n=17), ADH2_grad (n=13), MECH2_grad (n=18)*  *Mean test scores and standard deviations were:*   - *AD (M = 139.1290, SD = 13.65948).* - *ADH2_ grad (M = 226.2675, SD = 31.56533).* - *MECH2_ grad (372.9500, SD = 25.69106).*   *We conducted a one-way ANOVA to compare the means of the three groups.*  *A one-way ANOVA revealed a significant effect of the reactors on biogas production under instantaneous hydrogen addition, F(2,* *45) = 4.630, p =0.015. The effect size, eta squared (η²), was ).949, indicating a large effect.*  *Games-Howell post hoc test showed the MECH2_grad scored higher than both AD (p < 0.001) and ADH2_grad (p < 0.001) groups. The ADH2_grad reactor scored higher than the AD (p < 0.001).*  *These findings suggest that MECH2_inst leads to the highest methane yield under gradual hydrogen addition, followed by ADH2_grad, and lastly, AD. The effect size confirms these differences are practically significant.* |
| Methane yield | Instantaneous | *This study compared the effects of various systems on methane yield under instantaneous hydrogen addition. AD (n=17), ADH2_inst (n=21), MECH2_inst(n=11)*  *Mean test scores and standard deviations were:*   - *AD (M = 139.1290, SD = 13.65948).* - *ADH2_inst (M = 31.2550, SD = 54.54918).* - *MECH2_inst (M = 358.4411, SD = 30.57175).*   *We conducted a one-way ANOVA to compare the means of the three groups.*  *A one-way ANOVA revealed a significant effect of the reactors on biogas production under instantaneous hydrogen addition, F(2,* *46) = 11.429, p < 0.001. The effect size, eta squared (η²), was 0.915, indicating a large effect.*  *Games-Howell post hoc test showed the MECH2_inst scored higher than both AD (p < 0.001) and ADH2_inst (p < 0.01) groups. The AD reactor scored higher than the ADH2_inst (p < 0.05).*  *These findings suggest that MECH2_inst leads to the highest methane yield under instantaneous hydrogen addition, followed by AD, and lastly, ADH2_inst. The effect size confirms these differences are practically significant.* |
| pH | Gradual | *This study compared the effects of various systems on pH under gradual hydrogen addition. AD(n=7), ADH2_grad (n=22), MECH2_grad (n=22)*  *Mean test scores and standard deviations were:*   - *AD (M = 6.4686, SD = 0.11067).* - *ADH2_ grad (M = 6.5000, SD = .00000).* - *MECH2_ grad (M = 6.7000, SD = .00000).*   *We conducted a one-way ANOVA to compare the means of the three groups.*  *A one-way ANOVA revealed a significant effect of the reactors on biogas production under instantaneous hydrogen addition, F(2,* *48) = 55.073, p < 0.001. The effect size, eta squared (η²), was 0.881, indicating a large effect.*  *Games-Howell post hoc test showed the MECH2_grad scored higher than both AD (p =0.004) and ADH2_grad (p < 0.001) groups. There was no meaningful difference between ADH2_grad reactor and the AD (p =0.744).*  *These findings suggest that MECH2_inst leads to the highest pH under gradual hydrogen addition. The effect size confirms these differences are practically significant.* |
| pH | Instantaneous | *This study compared the effects of various systems on methane yield under instantaneous hydrogen addition. AD(n=7), ADH2_inst (n=9), MECH2_inst (n=7)*  *Mean test scores and standard deviations were:*   - *AD (M = 6.4686, SD = 0.11067).* - *ADH2_inst (M = 5.8667, SD =0 .67700).* - *MECH2_inst (M = 6.6000, SD = 0.00).*   *We conducted a one-way ANOVA to compare the means of the three groups.*  *A one-way ANOVA revealed a significant effect of the reactors on biogas production under instantaneous hydrogen addition, F(2,* *20) = 22.975, p < 0.001. The effect size, eta squared (η²), was 0.401, indicating a large effect.*  *Games-Howell post hoc test showed the MECH2_inst scored higher than both AD (p =* *.046) and ADH2_inst (p = 0.028) groups. There was no meaningful difference between AD reactor and the ADH2_inst (p =* 0*.067).*  *These findings suggest that MECH2_inst leads to the highest pH under instantaneous hydrogen addition. The effect size confirms these differences are practically significant.* |
| Propionate | Gradual | *This study compared the effects of various systems on effluent propionate under gradual hydrogen addition. AD (n=7), ADH2_grad (n=10), MECH2_grad(n=10)*  *Mean test scores and standard deviations were:*   - *AD (M = 119.2857, SD = 57.60994).* - *ADH2_ grad (M =* *66.6700, SD =* *15.55764).* - *MECH2_ grad (M= 62.3770, SD = 12.02255).*   *We conducted a one-way ANOVA to compare the means of the three groups.*  *A one-way ANOVA revealed a significant effect of the reactors on biogas production under instantaneous hydrogen addition, F(2,* *24) = 6.449, p =* 0*.006. The effect size, eta squared (η²), was 0.401, indicating a large effect.*  *Games-Howell post hoc test showed there was no meaningful difference between the reactors. In all cases P value was greater than 0.005 (p> 0.05)*  *These findings suggest that under gradual hydrogen addition there was not a significant difference between effluent propionate of the systems.* |
| Propionate | Instantaneous | *This study compared the effects of various systems on methane yield under instantaneous hydrogen addition. AD (n=7), ADH2_inst (n=9), MECH2_inst (n=9)*  *Mean test scores and standard deviations were:*   - *AD (M = 119.2857, SD = 57.60994).* - *ADH2_inst (M = 786.2772, SD =* *905.64582).* - *MECH2_inst (M = 80.2324, SD = 52.64023).*   *We conducted a one-way ANOVA to compare the means of the three groups.*  *A one-way ANOVA revealed a significant effect of the reactors on effluent propionate under instantaneous hydrogen addition, F(2,* *22) = 36.132, p < 0.001. The effect size, eta squared (η²), was 0* *.293, indicating a large effect.*  *Games-Howell post hoc test showed there was not a meaningful difference between MECH2_inst and AD (p =* 0*.889)*  *The ADH2_inst reactor had the highest propionate compared to both AD (p = 0* *.024) and MECH2_inst (p =* 0 *.012). The effect size confirms these differences are practically significant.* |
| Acetate | Gradual | *This study compared the effects of various systems on effluent acetate under gradual hydrogen addition. AD (n=8), ADH2_grad(n=10), MECH2_grad (n=10)*  *Mean test scores and standard deviations were:*   - *AD (M = 89.7299, SD = 21.74054).* - *ADH2_ grad (M =* *133.5980, SD =* *45.89480).* - *MECH2_ grad (M= 100.1000, SD = 36.20301).*   *We conducted a one-way ANOVA to compare the means of the three groups.*  *Levene's test was not significant (p > .05), indicating equal variances across groups. A one-way ANOVA revealed a significant effect of the reactors on biogas production under instantaneous hydrogen addition, F(2,* *25) = 2.596, p =* .095*. The effect size, eta squared (η²), was 0* *.224, indicating a large effect.*  *Games-Howell post hoc test showed there was no meaningful difference between the reactors. In all cases P value was greater than 0.005 (p> 0.05)*  *These findings suggest that under gradual hydrogen addition there was not a significant difference between effluent acetate of the systems.* |
| Acetate | Instantaneous | *This study compared the effects of various systems on effluent acetate under instantaneous hydrogen addition. AD (n=9), ADH2_inst (n=9), MECH2_inst (n=9)*  *Mean test scores and standard deviations were:*   - *AD (M = 87.5376, SD = 21.37338).* - *ADH2_inst (M = 140.2014, SD =* *56.69909).* - *MECH2_inst (M = 66.4019, SD = 24.20651).*   *We conducted a one-way ANOVA to compare the means of the three groups.*  *A one-way ANOVA revealed a significant effect of the reactors on effluent propionate under instantaneous hydrogen addition, F(2,* *24) = 9.344, p < 0.001. The effect size, eta squared (η²), was 0.433, indicating a large effect.*  *Games-Howell post hoc test showed the MECH2_inst reactor scored lower than ADH2_inst for effluent acetate (p =* 0*.011).*  *was not a meaningful difference between MECH2_inst and AD (p =* 0*.154) .The effect size confirms these differences are practically significant.* |

Figure 1 One-way ANOVA results - Effluent acetate under gradual hydrogen addition

Figure 2 One-way ANOVA results - Effluent acetate under instantaneous hydrogen addition

Figure 3 One-way ANOVA results - Biogas production under instantaneous hydrogen addition

Figure 4 One-way ANOVA results Biogas production under instantaneous hydrogen addition

Figure 5 One-way ANOVA results - Methane content under gradual hydrogen addition

Figure 6 One-way ANOVA results - Methane content under instantaneous hydrogen addition

Figure 7 One-way ANOVA results - Methane yield under gradual hydrogen addition

Figure 8 One-way ANOVA results - Methane yield under instantaneous hydrogen addition

Figure 9 One-way ANOVA results - pH variations under gradual hydrogen addition

Figure 10 One-way ANOVA results - pH variations under instantaneous hydrogen addition

Figure 11 One-way ANOVA results - Effluent propionate under instantaneous hydrogen addition

Figure 12 One-way ANOVA results - Effluent propionate under gradual hydrogen addition


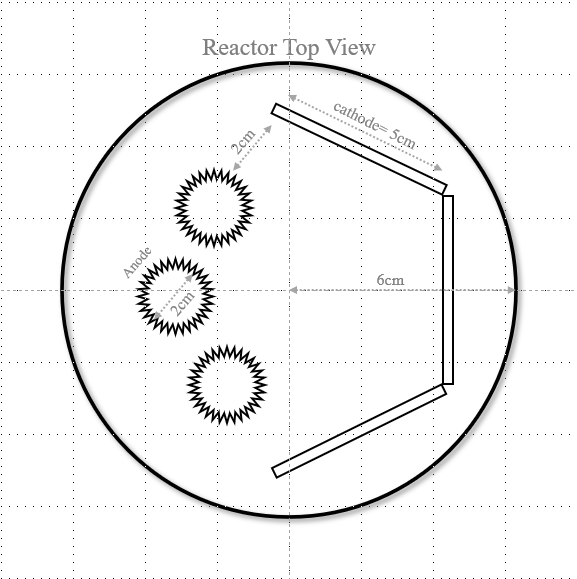


Figure 13 Microbial Electrolysis cell reactor top-view showing configuration of anodes and cathodes


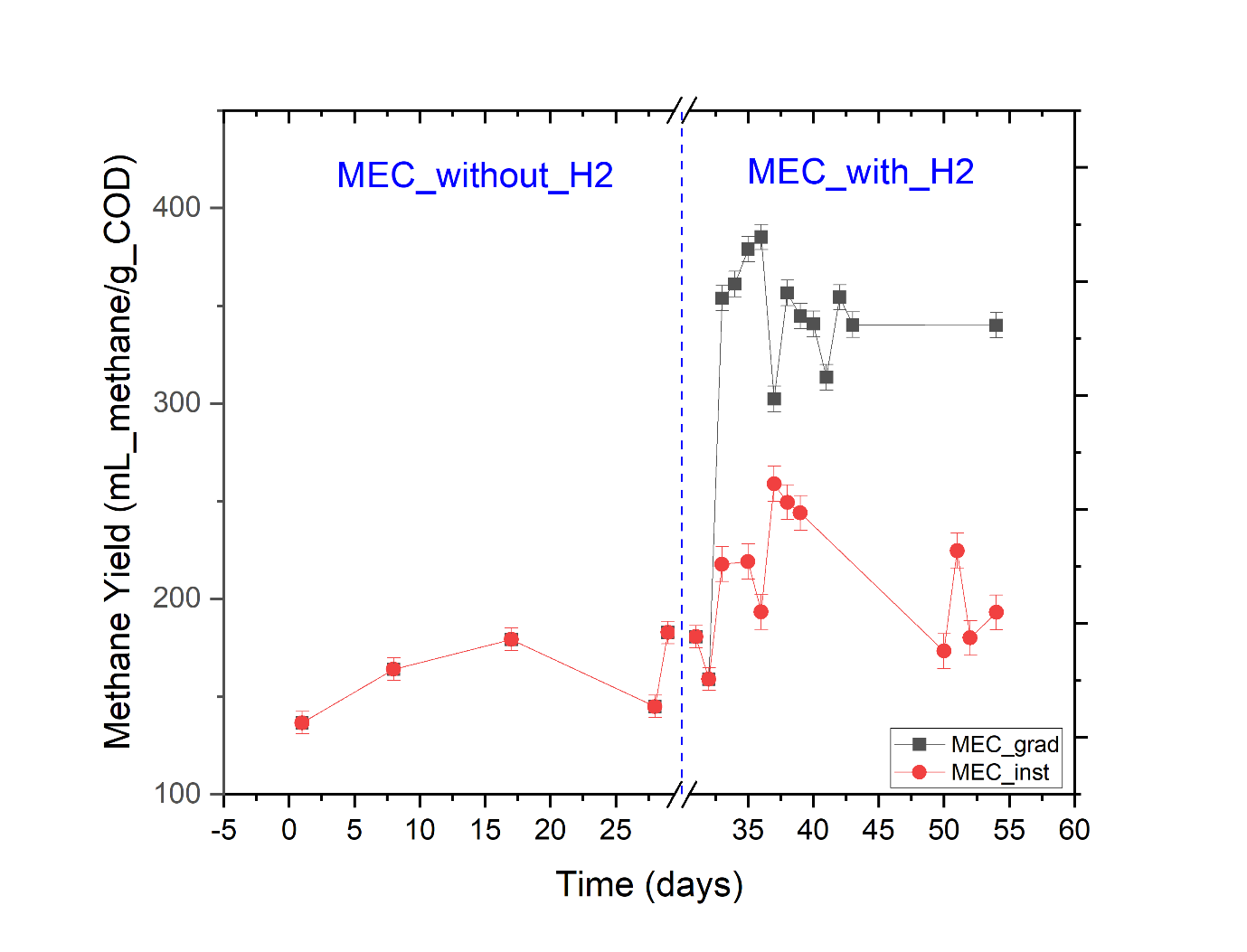


Figure 14 Methane yield (MEC with and without Hydrogen)

Table 2 Comparison of Reactor configuration and results of biological hydrogen methanation system

|  | **H2 injection levels** | **Temperature** | **HRT** | **Volume of reactor** | **Type of reactor** | **Injection mode (instantaneousvs gradual)** | **MEC integrated?** | **pH drop and VFA accumulation** | **reference** |
| --- | --- | --- | --- | --- | --- | --- | --- | --- | --- |
| 1 | 4:1 (4 m3H2= 1 m3 CO2) | Mesophilic |  | 1000 m3 | Theoretical modelling | Not mentioned | no | Could theoretically occur | (Bensmann et al. 2014) |
| 2 | 4:1 and lower (1.5:1) | mesophilic | 30 days | 20 L | CSTR | Not mentioned | no | Slight increase in VFA concentraion at rate of 4:1 and 1:1, H2:CO2 were in the stoichiometric range | (Cazaudehore et al. 2025) |
| 3 | 4:1 | Thermophilic | Not mentioned | 10L | continuous | gradual | no | Not measured | (Buffiere et al. 2025) |
| 4 | 4:1 | mesophilic | 5.47 – 10.96 s | 1.5 L | continuous | Continuous | no | No direct measurement | (Haitz et al. 2024) |
| 5 | Not explicitly mentioned | mesophilic | Not mentioned | 4-6.5 L | continuous | continuous | no | Acetate accumulation between 456.42 and 5221.42 mg/L) | (Savvas et al. 2024) |
| 6 | Not explicitly mentioned | mesophilic | 12 hours | 5 | Upflow anaerobic sludge blanket (UASB) | Continuous mode (1-10 mL/min) intermittent mode (20 min on - 20-40 min off) | no | pH dropped due in M4 due to excess H2 and potential VFA accumulation | (Liu et al. 2024) |
| 7 | 4:1 | mesophilic | 30 days | 0.15 L | Batch Reactors | Batch injection (into the headspace) | no | Acetic acid accumulation was obsaerved (up to 431.2 mg/L and lasted 1 -5 days) 9pure hydrogen a10ddition inhi11bited methanogenesis due to VFA buildup | (Zheng et al. 2023) |
| 8 | N/A (hydrogen production occurred) | mesophilic | 40 -33 days | 20L | Pilot scale semi continuous AD | N/A | no | Propionic acid accumulation at 8g/L at high hydrogen pressure due to hydrogen production in the system. Consequently methane production declined constantly | (Rocamora et al. 2023) |
| 9 | 4:1 | thermophilic | 10 days | 6L | CSTR | continuous | no | Acetic acid accumulation observed under high pressure up to 740mg/L | (Li 2022) |
| 10 | <4:1 | mesophilic | 18 days | 120 mL | batch | instantaneous | no | VFA accumulation occurred while exogenously added h2 was present in the headspace (up tpo propionate : 1.10 ± 0.16 mmol1 × L− 1 and acetate 12approx. 1.27 ± 0.06 mmol1 × L− 1) | (Vechi et al. 2021) |
| 11 | 1:1 and 4:1 | mesophilic | 25 days | 11.6L | cstr | continuous | no | Up to 1600 mg/L at 4:1 ratio (7.2 mL/min hydrogen) | (Zhu et al. 2020) |
| 12 | 4:1 | mesophilic | Not mentioned | 600 mL | Stirred reactors | continuous | yes | Not measured | (Park et al. 2020) |
| 13 | < and equal 4:1 | Meso and thermo | 25 days | 11.2 L | Intermittent and continuous mixing | Continuous injection | no | Acetate and propionate accumulation up to VFA of 2026 mg/L in meso and 2400 mg/L for thermo | (Zhu et al. 2019b) |
| 14 | 1:1 and 4:1 | thermophilic | 25 days | 11.2 | Intermittent and continuous mixing | Continuous injection | no | VFA accumulation in form of acetate and propionate up to VFA= 3317+326 mg/L | (Zhu et al. 2019a) |
| 15 | 4:1 | mesophilic | 20 days | 20L | Gas recirculation mode used | continuous | no | Reported low hydrogen addition rate and high gas recirculation avoided VFA accumulation | (Alfaro et al. 2019) |
| 16 | <4:1 | mesophilic | Not directly reported | Not directly reported | Tubular biotrickling filter reactor | continuous | no | pH drop and methane production reduction (did not directly report VFAs) | (Dupnock and Deshusses 2017) |
| 17 | 2:1 – 10:1 | mesophilic | 20-23 days | 2L | Stirred reactors | Pulse injection into the headspace | no | VFA accumulation at high hydrogen rates up to 1182+112 | (Agneessens et al. 2017) |
| **18** | **>4:1** | **mesophilic** | **30 days** | **1.5L** | **CSTR** | **Gradual vs instantaneous** | **Yes** | - **No VFA Accumulation under gradual injection** - **System failure under instantaneous hydrogen addition expect for MECs** | **Current Study** |

References

Agneessens LM, Ottosen LDM, Voigt NV, Nielsen JL, de Jonge N, Fischer CH, Kofoed MVW (2017) In-situ biogas upgrading with pulse H2 additions: The relevance of methanogen adaption and inorganic carbon level. Bioresour Technol 233:256-263. doi:<https://doi.org/10.1016/j.biortech.2017.02.016>

Alfaro N, Fdz-Polanco M, Fdz-Polanco F, Diaz I (2019) H2 addition through a submerged membrane for in-situ biogas upgrading in the anaerobic digestion of sewage sludge. Bioresour Technol 280:1-8. doi:<https://doi.org/10.1016/j.biortech.2019.01.135>

Bensmann A, Hanke-Rauschenbach R, Heyer R, Kohrs F, Benndorf D, Reichl U, Sundmacher K (2014) Biological methanation of hydrogen within biogas plants: a model-based feasibility study. Applied energy 134:413-425. doi:<https://doi.org/10.1016/j.apenergy.2014.08.047>

Buffiere P, Ramirez DA, Franco RT, Figueras J, Hattou S, Benbelkacem H (2025) Oxygen traces impact on biological methanation from hydrogen and CO(2). Bioresour Technol 419:132080. doi:<https://doi.org/10.1016/j.biortech.2025.132080>

Cazaudehore G, Peyrelasse C, Monlau F, Castel L, Leonardi F, Guyoneaud R, Sambusiti C (2025) Optimization of hydrogen dosage for enhanced in-situ bio-methanation. Bioresource Technology Reports 31. doi:<https://doi.org/10.1016/j.biteb.2025.102245>

Dupnock TL, Deshusses MA (2017) High-performance biogas upgrading using a biotrickling filter and hydrogenotrophic methanogens. Applied biochemistry and biotechnology 183:488-502. doi:<https://doi.org/10.1007/s12010-017-2569-2>

Haitz F, Jochum O, Lasota A, Friedrich A, Bieri M, Stalder M, Schaub M, Hochberg U, Zell C (2024) Continuous Biological Ex Situ Methanation of CO2 and H2 in a Novel Inverse Membrane Reactor (IMR). Processes 12 (10). doi:<https://doi.org/10.3390/pr12102305>

Li D (2022) Model application to a lab-scale thermophilic hydrogenotrophic methanation system. Biochemical Engineering Journal 177:108228. doi:<https://doi.org/10.1016/j.bej.2021.108228>

Liu S, Ma X, Yao S, Zhu X, Ma Y, Chen Z, Liang J (2024) Anaerobic Digestion Enhancement of Brewery Sludge Assisted by Exogenous Hydrogen. BioEnergy Research 17 (3):1943-1952. doi:<https://doi.org/10.1007/s12155-024-10758-z>

Park J-G, Heo T-Y, Kwon H-J, Shi W-Q, Jun H-B (2020) Effects of voltage supply on the methane production rates and pathways in an anaerobic digestion reactor using different electron donors. International Journal of Hydrogen Energy 45 (16):9459-9468. doi:<https://doi.org/10.1016/j.ijhydene.2020.01.237>

Rocamora I, Wagland ST, Hassard F, Villa R, Peces M, Simpson EW, Fernández O, Bajón-Fernández Y (2023) Inhibitory mechanisms on dry anaerobic digestion: ammonia, hydrogen and propionic acid relationship. Waste Management 161:29-42. doi:<https://doi.org/10.1016/j.wasman.2023.02.009>

Savvas S, Gangappa R, Ni X-W, Davies W, Barton W, Thomason M, Patterson T, Esteves SR (2024) The tubular baffled reactor and its potential for the biological methanation of carbon dioxide. Renewable Energy 232:121053. doi:<https://doi.org/10.1016/j.renene.2024.121053>

Vechi NT, Agneessens LM, Feilberg A, Ottosen LDM, Kofoed MVW (2021) In situ biomethanation: Inoculum origin influences acetate consumption rate during hydrogen addition. Bioresource Technology Reports 14. doi:<https://doi.org/10.1016/j.biteb.2021.100656>

Zheng M, Ou H, Dong F, He C, Hu Z, Wang W (2023) Mechanism insights into enhanced treatment of wasted activated sludge by hydrogen-mediated anaerobic digestion. Environmental Science and Pollution Research 30 (16):47787-47799. doi:<https://doi.org/10.1007/s11356-023-25657-7>

Zhu X, Cao Q, Chen Y, Sun X, Liu X, Li D (2019a) Effects of mixing and sodium formate on thermophilic in-situ biogas upgrading by H2 addition. Journal of Cleaner Production 216:373-381. doi:<https://doi.org/10.1016/j.jclepro.2019.01.245>

Zhu X, Chen L, Chen Y, Cao Q, Liu X, Li D (2019b) Differences of methanogenesis between mesophilic and thermophilic in situ biogas-upgrading systems by hydrogen addition. J Ind Microbiol Biotechnol 46 (11):1569-1581. doi:<https://doi.org/10.1007/s10295-019-02219-w>

Zhu X, Chen L, Chen Y, Cao Q, Liu X, Li D (2020) Effect of H2 addition on the microbial community structure of a mesophilic anaerobic digestion system. Energy 198. doi:<https://doi.org/10.1016/j.energy.2020.117368>
